# From *in planta* Function to Vitamin-Rich Food Crops: The ACE of Biofortification

**DOI:** 10.3389/fpls.2018.01862

**Published:** 2018-12-18

**Authors:** Simon Strobbe, Jolien De Lepeleire, Dominique Van Der Straeten

**Affiliations:** Laboratory of Functional Plant Biology, Department of Biology, Ghent University, Ghent, Belgium

**Keywords:** vitamin metabolism, crop improvement, hidden hunger, malnutrition, plant development, carotenoids, ascorbate, tocochromanols

## Abstract

Humans are highly dependent on plants to reach their dietary requirements, as plant products contribute both to energy and essential nutrients. For many decades, plant breeders have been able to gradually increase yields of several staple crops, thereby alleviating nutritional needs with varying degrees of success. However, many staple crops such as rice, wheat and corn, although delivering sufficient calories, fail to satisfy micronutrient demands, causing the so called ‘hidden hunger.’ Biofortification, the process of augmenting nutritional quality of food through the use of agricultural methodologies, is a pivotal asset in the fight against micronutrient malnutrition, mainly due to vitamin and mineral deficiencies. Several technical advances have led to recent breakthroughs. Nutritional genomics has come to fruition based on marker-assisted breeding enabling rapid identification of micronutrient related quantitative trait loci (QTL) in the germplasm of interest. As a complement to these breeding techniques, metabolic engineering approaches, relying on a continuously growing fundamental knowledge of plant metabolism, are able to overcome some of the inevitable pitfalls of breeding. Alteration of micronutrient levels does also require fundamental knowledge about their role and influence on plant growth and development. This review focuses on our knowledge about provitamin A (beta-carotene), vitamin C (ascorbate) and the vitamin E group (tocochromanols). We begin by providing an overview of the functions of these vitamins *in planta*, followed by highlighting some of the achievements in the nutritional enhancement of food crops via conventional breeding and genetic modification, concluding with an evaluation of the need for such biofortification interventions. The review further elaborates on the vast potential of creating nutritionally enhanced crops through multi-pathway engineering and the synergistic potential of conventional breeding in combination with genetic engineering, including the impact of novel genome editing technologies.

## Introduction

Ensuring food security to all populations is considered a top priority for global societal progress. Undernourishment has dropped severely in the last decades, from roughly 20% of the world population in 1990 to little above 10% in 2016 (Food and Agriculture Organization [FAOSTAT], 2017). It stands undisputed that continuing efforts should be undertaken to further reduce the number of undernourished people in the world, which is still close to 800 million. The successful reduction of malnourishment can partly be attributed to the increase in staple crop yield witnessed over the last decades. Indeed, in the last 25 years, the production per hectare of rice, wheat and potato has risen by 30% (Food and Agriculture Organization [FAOSTAT], 2017). However, these crops often fail to supply adequate amounts of micronutrients, thereby augmenting the prevalence of micronutrient malnutrition (MNM, ‘hidden hunger’). These micronutrients include minerals such as iron, zinc, selenium, and manganese, as well as a wide range of vitamins (Miller and Welch, 2013). Hidden hunger affects an alarming two billion people (Bailey et al., 2015; Rautiainen et al., 2016), mostly in the form of anemia, occurring in one-fourth of the human population (McLean et al., 2009). The case of anemia clearly demonstrates the physiological impact of MNM, as its onset has been linked to deficiencies in different micronutrients such as iron, vitamin B1, B9, and B12 (Green, 2003; Imdad and Bhutta, 2012; Stabler, 2013). The importance of MNM is further highlighted by the large, calculated economic benefit a reduction of child malnutrition would have on development. Among 19 prioritized investment-for-development targets listed in the Post-2015 Consensus, the Copenhagen Consensus Center think-tank has ranked the reduction of child malnutrition as the human development investment with the highest potential economic returns (Copenhagen Consensus, 2012).

Vitamin deficiencies can be combatted by supplementation, industrial fortification, biofortification, and educational interventions encouraging dietary diversification. It should be noted that choice of the intervention strategy to be implemented depends on regional dietary and cultural differences (Bailey et al., 2015). However, some universally valid remarks can be made. Supplementation, whether by administration of (multi-)vitamin pills or by fortification of cereal products (mandatory in many countries), has shown to be a fast and powerful means to reduce vitamin deficiencies (Sandjaja et al., 2015; Atta et al., 2016; Wang et al., 2016). Unfortunately, this intervention is not easily applicable to poor rural populations in need (Blancquaert et al., 2014). Furthermore, supplementation could exhibit adverse effects, as demonstrated by the observation of increased mortality and higher risk of colorectal cancer in males upon vitamin A and B9 supplementation, respectively (Benn et al., 2015; Cho et al., 2015). Educational efforts, aimed to change the diet and/or processing of food by populations suffering from vitamin deficiencies, are an excellent way to fight MNM, tackling the root causes of the problem. However, these interventions are expensive and imply cultural and agronomical changes, the feasibility of which cannot be guaranteed (Low et al., 2007; Faber and Laurie, 2011). Biofortification, which consists of enhancing the natural vitamin level of food crops, is advocated as a powerful complementary method to fight vitamin malnutrition, circumventing the aforementioned obstructions (Fitzpatrick et al., 2012; Blancquaert et al., 2017; Saltzman et al., 2017).

Biofortification of local crops can be considered a sustainable and cost-effective means to reduce vitamin shortage (Meenakshi et al., 2010; De Steur et al., 2015). Two methods of biofortification, apart from agronomical interventions (Cakmak and Kutman, 2017; Watanabe et al., 2017), can be distinguished. First, biofortified crops can be obtained by conventional breeding or using molecular techniques, to obtain novel high-vitamin lines (Ortiz-Monasterio et al., 2007; Bouis and Saltzman, 2017). Unfortunately, this approach relies on the presence of sufficient variation of vitamin levels in sexually compatible germplasm collections (Shimelis and Laing, 2012; Strobbe and Van Der Straeten, 2017). Furthermore, introgression of a certain trait of interest into various region-specific crops demands time-consuming selection over several generations. Novel breeding techniques, however, enable more rapid retrieval of the desired trait via genome wide association mapping (GWAS) or accelerated selection of the introgression lines using marker-assisted breeding (MAB) (Borrill et al., 2014; Esuma et al., 2016). Second, metabolic engineering via GM-technology allows introduction of one or multiple genes of interest, influencing plant metabolism toward increased accumulation of the particular vitamin. As it is not dependent on sexual compatibility of gene source, genetic elements from a very diverse pool could be utilized, including the vast genetic diversity of prokaryotes. Moreover, metabolic engineering can be implemented in a time and tissue-specific manner via selection of promoters with the desired temporal and spatial characteristics. This method, however, demands prior knowledge about specific vitamin metabolism as well as availability of adequate promoters. In principle, it allows the creation of a model vitamin engineering strategy, which can be implemented in a variety of cultivars and crops. However, this cannot be generalized, due to differences in vitamin regulation and metabolism in different crops and tissues (Strobbe and Van Der Straeten, 2017). Interestingly, novel genome editing techniques such as the CRISPR/Cas system allow directed mutagenesis and editing of targeted genomic regions (Cong et al., 2013; Luo et al., 2016), enabling targeted metabolic engineering approaches, though still constrained by the limitation of genetic diversity of the engineered species. A combination of the aforementioned techniques, could offer powerful solutions to alleviate vitamin deficiencies.

Biofortification should be carried out with due consideration to its effects on the plant’s physiology and not only with the consumers’ vitamin needs in mind. The health impact of a biofortified crop could be region specific, due to genetic, environmental and dietary factors. Massive consumption of staple crops with low content of one or more micronutrients appears to be a major factor aggravating the incidence of the deficiency. Therefore, biofortification of these crops is advised. Biofortification endeavors should, however, not solely focus on vitamin content, but take all factors influencing vitamin-specific nutritional value of the particular crop into considerations, such as storage and processing stability, as well as bioavailability (Fitzpatrick et al., 2012; Blancquaert et al., 2015; Diaz-Gomez et al., 2017b).

The three vitamins covered in this review–namely vitamin A, C and E–have been the subject of various biofortification approaches due to their impact on human health and very low content in the six major staple crops consumed worldwide (Table 1). But because of their roles in key enzymatic and stress-related stress response roles, there is a need to bundle the existing knowledge of *in planta* vitamin metabolism, taking possible detrimental effects on crop growth into consideration. Consequently, proper design of metabolic engineering approaches for vitamin biofortification requires a profound understanding of *in planta* vitamin biosynthesis as well as its metabolism.

**Table 1:** Vitamin A, C, E content and highest consumption of six major staple crops.

	Wheat (*Triticum aestivum*) (soft white)	Rice (*Oryza sativa*) (white, long-grain, regular, raw, unenriched)	Potato (*Solanum tuberosum*) (flesh and skin, raw)	Cassava (*Manihot esculenta*) (raw)	Corn (*Zea mays*) (sweet, white, raw)	Plantain (*Musa* sp.) (raw)	RDA^1^
provitamin A RAE^2^ (μg/100g)	2.7 (80)	0 (>100)	0.6 (>100)	3.9 (50)	0.3 (>100)	338.1 (±sufficient)	1300
Ascorbate (vitamin C) (μg/100g)	0 (>100)	0 (>100)	19,700 (2)	20,600 (sufficient)	6,800 (5)	18,400 (8)	120,000
α-tocopherol (vitamin E) (μg/100 g)	1,010 (4)	110 (37)	10 (>100)	190 (15)	70 (63)	140 (39)	19,000
Highest consumption (g/capita.day)^4^	609 (Azerbaijan)	470^3^ (Bangladesh)	502 (Belarus)	678 (Democratic Republic of the Congo)	434 (Lesotho)	350 (Ghana)	

*Data were retrieved from the USDA database (United States Department of Agriculture [USDA], 2016). RDA data were retrieved from (Trumbo et al., 2001). Values represent vitamin content of 100 g fresh edible portion of the crop product. Fold enhancement needed to reach the RDA of the corresponding vitamin upon consumption of the highest average national servings are indicated between brackets. Data on staple crop consumption are derived from FAOSTAT (Food and Agriculture Organization [FAOSTAT], 2017). Note that values of enhancement do not take bioavailability and processing losses into account, and though depending on the food matrix, can easily amount to 50% each*. *^*1*^Highest recommended daily allowance (RDA) is depicted (μg/day). ^*2*^Retinol activity equivalent. ^*3*^Milled equivalent. ^*4*^in 2013.*

In the past decades, major advances have been accomplished in biofortification of different food crops. Fortunately, some of these are already being used to combat MNM. However, the use of metabolically engineered, biofortified crops has not been implemented to date. Interestingly, the imminent commercialization of provitamin A-rich ‘Golden Rice’ might open doors toward application of other engineered biofortified crops. In this review, the incidence and pathophysiology of the different vitamin deficiencies are discussed, alongside with the status of knowledge on plant vitamin biosynthesis and physiology and the advances made in crop biofortification with these vitamins.

## Provitamin A – Carotenoids

**FIGURE 1:**
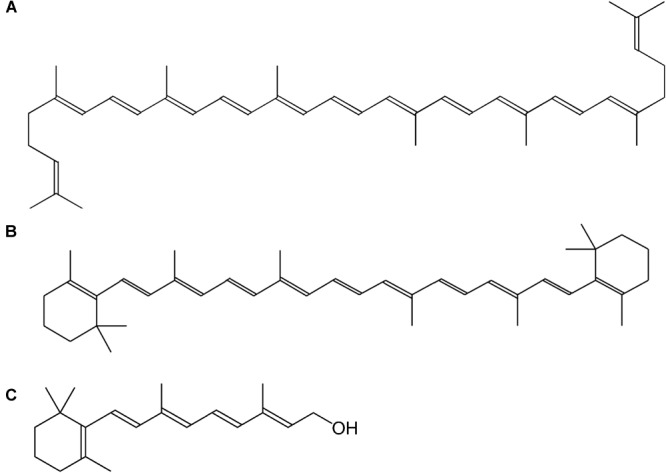
Chemical structure of vitamin A and its precursors. **(A)** Lycopene, **(B)** β-carotene, **(C)** all-*trans*-retinol.

Vitamin A is a collective term for different fat-soluble retinoid molecules (Bai et al., 2011), defined as every chemical structure able to fulfill the biological activity of all-*trans*-retinol (Figure 1C) upon human consumption (Eitenmiller et al., 2016). Carotenoids, comprise over 600 different compounds, only three of which can be metabolically converted to active vitamin-A substances such as retinol (Figure 1) and its oxidized equivalents retinal and retinoic acid (Asson-Batres and Rochette-Egly, 2016). Carotenoids represent the major source of provitamin A in the diet and are present throughout the plant kingdom. The general backbone is formed by head-to-tail linking of eight isoprene units, resulting in a C_40_-unsaturated chain, lycopene (Figure 1A), a carotenoid precursor (Eitenmiller et al., 2016). The most important carotenoid, β-carotene (Figure 1B), harbors cyclized β-ionone rings on both ends of the C_40_-chain (Figure 1). Because these molecules consist of long-chain conjugated polyene units, they are sensitive to oxidation, light, heat and acids (Asson-Batres and Rochette-Egly, 2016). Their sensitivity to oxidation, however, enables them to serve as antioxidants in plants and animals, as the radical resulting from interaction with reactive oxygen species (ROS), is much less hazardous by stabilization of the polyene groups. Vitamin A function, however, greatly exceeds its antioxidant properties, as it plays multiple roles in plant and animal physiology.

### Vitamin A Biosynthesis

The principal provitamin A for humans is β-carotene, which is composed of two symmetrical retinyl groups. One such retinyl group consists of a retinyl isoprenoid chain and a β-ionone ring which is important for vitamin A action (Figure 1) (Send and Sundholm, 2007). Hence, as α-carotene, γ-carotene and β-cryptoxanthin also carry 1 β-ionone ring, they possess 50% vitamin A activity. Provitamin A is synthesized in plastids in all photosynthetic organisms by enzymes associated with the thylakoid membrane, namely phytoene desaturase (PDS), ζ-carotene desaturase (ZDS), lycopene-β-cyclase (β-LCY) and lycopene-𝜀-cyclase (𝜀-LCY); or associated in multienzyme complexes (Cunningham and Gantt, 1998).

**FIGURE 2:**
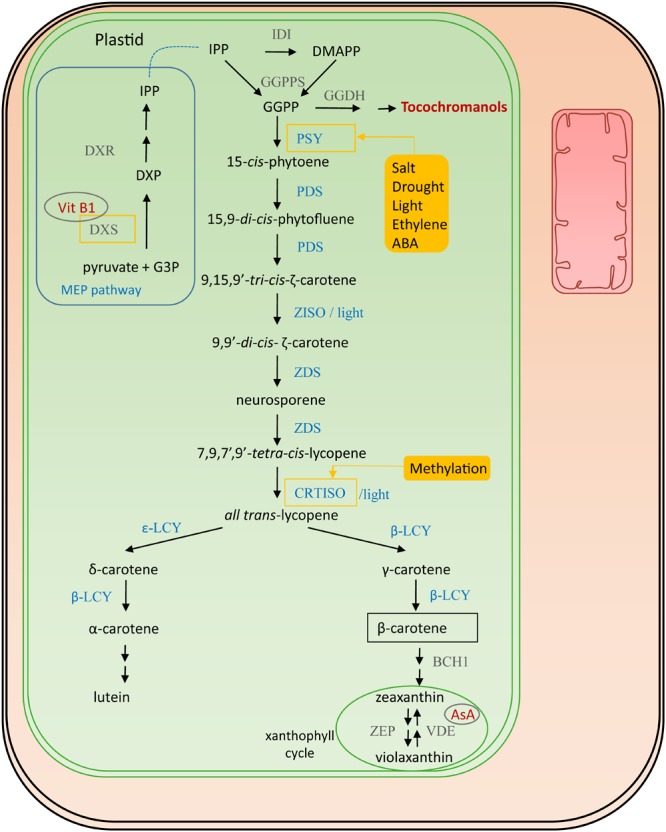
Provitamin A biosynthesis. Enzymes involved in its biosynthesis are marked in blue. Connections to other vitamin pathways are indicated in red. Filled yellow boxes indicate the external influences on the biosynthesis, affected enzymes surrounded by a yellow square. The regulatory influences on DXS are derived from studies on Arabidopsis (Estevez et al., 2001), those on PSY from studies in maize, rice and tomato (Li F.Q. et al., 2008; Welsch et al., 2008). Cofactors are encircled in gray. Abbreviations (in order of appearance in the pathway): G3P, glyceraldehyde-3-phosphate; DXS, 1-deoxy-D-xylulose-5-phosphate synthase; DXP, 1-deoxy-D-xylulose-5-phospate; DXR, DXP reductoisomerase; IPP, isopentenyl diphosphate isomerase; IDI, isopentenyl diphosphate isomerase; DMAPP, dimethylallyl diphosphate; GGPPS, geranylgeranyl diphosphate synthase; GGPP, geranylgeranyl diphosphate; PSY, phytoene synthase; PDS, phytoene desaturase; ZISO, ζ-carotene isomerase; ZDS, ζ-carotene desaturase; CRTISO, carotene isomerase; β-LCY, lycopene-β-cyclase; 𝜀-LCY, lycopene-𝜀-cyclase; BCH1, β-carotene hydroxylase; ZEP1, zeaxanthin epoxidase; VDE, violaxanthin de-epoxidase; AsA, ascorbate.

The direct precursor for provitamin A is geranylgeranyl diphosphate (GGPP) (see also vitamin E biosynthesis, 4.1), which is formed by the condensation of the building blocks isopentenyl diphosphate (IPP) and 3 dimethylallyl diphosphate (DMAPP) molecules, by GGPP synthase (GGPPS) (Ruiz-Sola et al., 2016) (Figure 2). IPP is produced in the plastid-localized 2-C-methyl-D-erythritol 4-phosphate (MEP) pathway and DMAPP is its isomerisation product catalyzed by isopentenyl diphosphate isomerase (IDI). GGPP is also the precursor for chlorophylls, ubiquinones, tocopherols, gibberellins and terpenoids (Saini et al., 2015). The first step of the actual provitamin A biosynthetic pathway is the condensation of two GGPP molecules by phytoene synthase (PSY) forming 15-*cis*-phytoene, assumed to be a rate-limiting step (Fray and Grierson, 1993; Li F.Q. et al., 2008; Cazzonelli and Pogson, 2010). In most plant species multiple redundant *PSY* genes are present which are differentially regulated. Salt and drought, are environmental factors which induce *PSY* expression, thereby enhancing carotene levels (Ruiz-Sola et al., 2014; Nisar et al., 2015). Moreover, ethylene is known to have a positive influence on accumulation of carotenoids, inducing *PSY* expression (Zhang et al., 2018). This aspect is particularly important in fruit ripening and has therefore been studied in mango (*Mangifera indica*) (Ma et al., 2018), durian (*Durio zibethinus*) (Wisutiamonkul et al., 2017) and tomato (*Solanum lycopersicum*) (Su et al., 2015; Cruz et al., 2018). A recent study identified the tomato transcription factor SlCMB1 as a regulator of both ethylene production and carotenoid accumulation (via *PSY* and *PDS*) (Zhang et al., 2018). PSY can therefore, in most plants, be considered a master regulator of carotenoid accumulation, given that it is also stimulated by light, directly controlled by transcription factors PHYTOCHROME INTERACTING FACTOR 1 (PIF1) and LONG HYPOCOTYL 5 (HY5) in Arabidopsis photomorphogenesis (Toledo-Ortiz et al., 2010; Llorente et al., 2017). In the subsequent biosynthesis step, directly downstream of PSY, 15-*cis*-phytoene is transformed into 9,15,9′-*tri-cis*-ζ-carotene via a 15,9-*di-cis*-phytofluene intermediate by two consecutive desaturation reactions catalyzed by phytoene desaturase (PDS) (Pecker et al., 1992; Li et al., 1996; Qin et al., 2007). Subsequently, either a photoisomerization or an isomerization by ζ-carotene isomerase (ZISO) (Pecker et al., 1992; Li et al., 2007) results in 9,9′-*di*-*cis*-ζ-carotene. Reiteratively, two desaturation reactions are performed by ζ-carotene isomerase (ZDS) producing neurosporene followed by 7,9,7′,9′-*tetra-cis*-lycopene (prolycopene) (Wong et al., 2004; Dong et al., 2007). Finally, either light or carotene isomerase (CRTISO) isomerizes the *cis* bonds into *all trans*-lycopene. This enzyme is a secondary point of regulation, as it is epigenetically regulated via methylation (Cazzonelli et al., 2009). Several cyclization reactions result in the production of bicyclic carotenoids. Lycopene-β-cyclase (β-LCY) catalyzes the addition of β-ionone rings. One β-ionone ring leads to the formation of γ-carotene; a second one forms β-carotene. Lycopene-𝜀-cyclase (𝜀-LCY) catalyzes addition of 𝜀-ionone rings, forming δ-carotene. Addition of one β-ionone ring and an 𝜀-ionone ring on the other side of the linear backbone results in production of α-carotene. Essentially, the pathway bifurcates after lycopene synthesis into β,β- and 𝜀,β-carotenoids, and the relative activities of β-CLY and 𝜀-CLY determine the proportion of lycopene funneled to the two branches (Cazzonelli and Pogson, 2010). Hydroxylation of α-carotene gives rise to lutein, while hydroxylation of β-carotene leads to formation of zeaxanthin.

### Provitamin A Functions *in planta*

Oxygenated carotenoid derivatives are termed xanthophylls, whereas the non-oxygenated analogs are designated as carotenes. Distinct functions are attributed to these two classes of carotenoids.

#### Enhancing Light Harvesting and Photoprotection

Lipid soluble carotenoids play a major role in photoprotection. The conjugated double bonds in the carbon skeleton function as chromophore, allowing light absorption in the range of 450–570 nm, covering the absorption gap of chlorophyll. Consequently, they function as accessory pigments in photosynthesis, enhancing light harvesting in the blue–green spectral domain (Cogdell and Frank, 1987; Havaux et al., 2004), while also being required for the correct assembly of photosystems (Formaggio et al., 2001).

Xanthophylls are crucial in non-photochemical quenching (NPQ) of excess photon energy by thermal dissipation through molecular vibrations (Demmig-Adams and Adams, 1996). The xanthophyll cycle encompasses two antagonistic enzymes, violaxanthin de-epoxidase (VDE) which converts violaxanthin via antheraxanthin into zeaxanthin, and zeaxanthin epoxidase (ZEP) which performs the reversed reactions. This protective mechanism prevents the over-reduction of photosystem II (PSII) and the generation of ROS (Briantais, 1994). When the level of absorbed light exceeds the photochemical capacity of PSII, the acidification of the thylakoid lumen activates VDE. Additionally, ethylene was found to be a negative regulator of the cycle as it influences the activity and activation of VDE (Chen and Gallie, 2015). Overexpression of β-carotene hydroxylase (BCH1), causing a simultaneous increase in zeaxanthin and xanthophyll levels, enhances tolerance to high light and heat stress (Davison et al., 2002). The extra xanthophyll was shown to be associated with the PSII light-harvesting complexes (LHCII), and the plants exhibited reduced leaf necrosis and lipid peroxidation.

Carotenes are important to mitigate the generation of ROS during photosynthesis. Carotenoids can quench both triplet chlorophyll (^3^Chl^∗^) and singlet oxygen (^1^O_2_), protecting PSI and PSII from photoinhibition (Edge et al., 1997; Triantaphylides and Havaux, 2009). On the other hand xanthophylls like zeaxanthin are involved in the protection of the photosynthetic membranes against lipid peroxidation (Havaux and Niyogi, 1999; Davison et al., 2002).

#### Stress Signaling

Besides their role in photosynthesis, carotenoids perform a function in stress signaling, as stress-imposed singlet oxygen production can lead to a variety of oxidative cleavage carotenoid derivatives, several of which are reactive electrophile species (RES). One example of RES is the volatile β-cyclocitral (β-CC), which is capable of altering ^1^O_2_ responsive gene expression in relation to stress acclimation (Havaux, 2014). This RES-induced ^1^O_2_ response could interact with jasmonic acid (JA) signaling and thus compromise the JA-mediated responses to pathogens and herbivores in high light acclimated plants (Ramel et al., 2012). Another example in which carotenoid-derived signals are implicated in retrograde signaling resides in the control of chloroplast and leaf development. The albino Arabidopsis (*Arabidopsis thaliana*) null mutant of ZDS, Arabidopsis *zds/chloroplast biogenesis5 (clb5)*, exhibits abnormal leaf development and cell differentiation with weakened auxin responses. Introduction of the *pds3* mutation, compromising ζ-carotene synthesis, rescued the *clb5* mutant gene expression and leaf development phenotypes. This suggests that ζ-carotene isomers are implicated in regulating chloroplast biogenesis and leaf development (Avendano-Vazquez et al., 2014).

#### Shoot and Root Development

Inhibition of carotenoid production disturbs the rhythmic oscillation of the lateral root (LR) clock, necessary for establishment of pre-branch sites (Van Norman et al., 2014). The same decrease in LR capacity was observed when using an inhibitor of carotenoid cleavage dioxygenases (CCDs), but the carotenoid-derived signaling molecule responsible for the influence on root branching remains to be identified (Van Norman et al., 2014). Earlier mutant analysis has revealed the necessity for other carotenoid derived signals in normal development. The *bypass1* (*bps1*) mutant has short roots, a malfunctioning shoot apical meristem and leaf vasculature with an increasing severity in lower temperatures. Grafting experiments suggested the constitutive presence of a mobile root derived ‘bypass’ signal, which required β-carotene synthesis, but no CCDs. (Van Norman et al., 2004, 2014; Van Norman and Sieburth, 2007). *CAROTENOID CHLOROPLAST REGULATORY1* (*CCR1*) which encodes a histone methyltransferase Set Domain Group8 (SDG8), defines yet another link of carotenoids to shoot development. SDG8 is important for expression of *CRTISO* (Figure 2). Besides enhanced rosette growth and cauline branching, altered carotenoid content was observed in *ccr1* (Cazzonelli et al., 2009).

### Vitamin A in Human Health

#### Function and Pathophysiology of Vitamin A Deficiency

During the last decades, knowledge on vitamin A functioning in humans has greatly increased, emphasizing its tremendous clinical importance (Wiseman et al., 2017). Retinol and retinal vitamer forms of vitamin A play a pivotal role in proper function of vision and dark adaptation. Human vision depends on the regeneration of the vitamin A derivative 11-*cis*-retinal, necessary for the formation of rhodopsin (Tang et al., 2013; Hanneken et al., 2017; Tian et al., 2017). Rhodopsin in turn is required as pigment in the retinal receptor responsible for dark adaptation (Sommer, 2008; Wiseman et al., 2017). This explains why vitamin A deficiency (VAD) can lead to xerophthalmia, a pathophysiological condition of impaired vision, starting with night blindness, and ultimately leading to complete blindness due to corneal damage (Sommer, 2008; Chiu et al., 2016). Furthermore, vitamin A is known to have a beneficial impact on innate and adaptive immunity (Lima et al., 2010; Wiseman et al., 2017). Consequently, VAD induces increased susceptibility toward a variety of infections, particularly gastro-intestinal conditions (Brown and Noelle, 2015). Anemia, the most prevalent of all micronutrient deficiency-induced disorders, has also been linked to VAD, as vitamin A is able to influence iron metabolism (Semba et al., 1992; West et al., 2007). Human reproduction also depends on vitamin A, more particularly retinoic acid, as it is shown to be necessary in spermatogenesis as well as for proper embryo growth (Hogarth and Griswold, 2010; Clagett-Dame and Knutson, 2011; Wiseman et al., 2017). The above is, however, but a selection of the vast impact of vitamin A in all its vitamer entities on basic human physiology. It also illustrates the urgency of cutting back VAD incidence on a global scale.

#### Global Burden of Vitamin A Deficiency

Occurrence of VAD can be explained by poor dietary diversification, likely caused by high consumption of staples with low vitamin content (Table 1). An estimated 250 million children of preschool age suffer from VAD (Wiseman et al., 2017; World Health Organization [WHO], 2018). Moreover, 250–500 thousand children develop VAD-induced full blindness each year, half of these cases resulting in death within a year (World Health Organization [WHO], 2018). As VAD is known to have a negative impact on the human immune system (Brown and Noelle, 2015), many infection-related deaths could also, at least partially, be attributed to low vitamin A status, indicating that the incidence of VAD-induced mortality is potentially underestimated. UNICEF reported that vitamin A supplementation programs are able to save 350 thousand children lives annually (Dalmiya and Palmer, 2007; Sommer and Vyas, 2012). Despite these efforts, coverage of the supplementation programs remains poor in many regions, explaining the persistent occurrence of VAD in these populations. Though VAD is much more prevalent in low-income countries (Bailey et al., 2015; Wiseman et al., 2017), there is also a great need to enhance (pro)vitamin A uptake on a global scale, given the existence of VAD-induced disorders in the developed world (Chiu et al., 2016).

#### Sources of Vitamin A

Provitamin A is present in animal as well as plant derived foods (Bai et al., 2011; Mody, 2017). Meat and dairy products are typically rich in retinyl esters, which can be metabolized to retinol in the human body (Bai et al., 2011). In plant-derived food sources on the other hand, the provitamin A content is represented by carotenoids, β-carotene being the most prevalent (Grune et al., 2010). β-carotene can be converted to retinal by the human β-carotene 15,15′–monooxygenase (Lindqvist and Andersson, 2002), which is typically absent in strictly carnivorous mammals. Richly colored fruit and vegetables are good sources of provitamin A. Examples of high provitamin A carotenoid containing crops are carrots, sweet potatoes, pumpkin, kale and spinach (Harrison, 2005). The food matrix within which the vitamin is delivered is also of great importance, as it determines bioavailability, demonstrated by the increasing portion of bioavailable provitamin A in orange juice upon pasteurization (Aschoff et al., 2015). As the provitamin A content of a food source can be the result of a whole array of provitamin A (mostly carotenoids) compounds, the vitamin content is often described as retinol activity equivalent (RAE). The RAE measures the amount of provitamin A expressed as having the same bioactive power of a certain amount of retinal, taking bioavailability into account. The highest recommended daily allowance is 1.3 mg for lactating females. As all major staples, with the exception of plantain (cooking banana, *Musa* sp.), are poor sources of provitamin A (Table 1), there is a strong case for raising its level in those crops (De Moura et al., 2016).

### Biofortification: Toward Higher Provitamin A Levels in Crops

#### Metabolic Engineering

Over the last decades tremendous efforts has been invested in the augmentation of provitamin A levels in different crops (Giuliano, 2017). *PSY*, responsible for the first committed step of carotenoid biosynthesis, has been pinpointed as rate-limiting step, thereby serving as an ideal candidate gene in biofortification strategies (Fitzpatrick et al., 2012). A well-known example is the genetically engineered Golden Rice (*Oryza sativa*) (Ye et al., 2000; Beyer et al., 2002; Paine et al., 2005), which has a yellow color, due to its high carotenoid nature. In Golden Rice (Ye et al., 2000), metabolic engineering was achieved via endosperm-specific induction of the daffodil (*Narcissus pseudonarcissus*) *PSY* and bacterial (*Erwinia uredovora*) *carotene desaturase* (*CRT*), representing the steps in carotenoid synthesis which are naturally not expressed in rice endosperm. The Golden Rice engineering strategy was later improved by replacing the daffodil-derived *PSY* by a maize ortholog showing a stronger enzymatic activity in rice than the originally used daffodil enzyme and thus leading to higher beta-carotene levels in the so called Golden Rice 2 (GR2) (Paine et al., 2005). The latter rice lines contain up to 3.7 mg/100 g dry weight (DW) carotenoids in the endosperm. GR2 delivers 50% of a child’s RDA of provitamin A in 72 g dry rice. On top of its ability to be deployed to minimize VAD, Golden Rice can be considered a solid proof-of-concept, enabling implementation of this metabolic engineering strategy in a range of crops. Indeed, adopting this strategy into *Zea mays* yielded maize kernels with 6 mg/100 g DW β-carotene (Naqvi et al., 2009), corresponding to a 112-fold increase in total carotenoid content over the WT corn variety used in this study. Also in wheat (*Triticum aestivum*), this strategy has led to a 10-fold increase in endosperm carotenoid levels, reaching almost 500 μg/100 g dry weight (Cong et al., 2009).

Interestingly, a one-gene metabolic engineering approach, overexpressing only *PSY*, has also led to several successfully biofortified crops. In canola (rapeseed, *Brassica napus*), *PSY* introduction yielded 50-fold increase in seed carotenoid content (Shewmaker et al., 1999). Similarly, carotenoid content of potato (*Solanum tuberosum*) was elevated (up to 3.5 mg/100 g DW) mostly caused by strongly enhanced β-carotene levels (up to 1.1 mg/100 g DW) (Ducreux et al., 2005). In cassava (*Manihot esculenta*), root specific ectopic expression of *PSY* resulted in carotenoid levels to be elevated 20-fold, reaching 2.5 mg/100 g DW (Sayre et al., 2011). Finally, a recent *cis*-genic *PSY*-overexpression engineering approach resulted in banana lines reaching up to 5.5 mg/100g DW β-carotene equivalent content of fruits (Paul et al., 2017).

A different one-gene approach has been applied in tomato fruit engineering (Rosati et al., 2000; Ralley et al., 2016), as this tissue harbors high expression of genes controlling biosynthesis of lycopene, such as the aforementioned *PSY*. Therefore, a carotenoid biosynthesis gene, downstream of lycopene was a more appropriate choice for biofortification of carotenoid content in tomato fruit (Rosati et al., 2000). The lycopene β-cyclase gene (β-LCY), catalyzing the cyclization of the lycopene molecule by introduction of the β-ionone rings yielding β-carotene (Cunningham et al., 1996) (Figures 1A,B), was engineered in tomato fruit, resulting in high β-carotene tomatoes (Rosati et al., 2000; D’Ambrosio et al., 2004; Ralley et al., 2016).

Single gene approaches, despite reaching satisfying levels of provitamin A, could be strengthened by introduction of additional genes, further increasing flux through the biosynthetic pathway. Indeed, further research in canola resulted in seeds with over 1,000-fold increase in β-carotene, reaching over 20 mg/100 g fresh weight (FW) (Fujisawa et al., 2009). This was accomplished by introduction of seven bacterial genes, highlighting the power of multiple gene engineering as well as the applicability of prokaryotic genes (Fujisawa et al., 2009; Bai et al., 2011). Similarly, in potato, combined tuber-specific boosting of PSY, PDS and β-LCY (Figure 2) generated ‘golden potato’ tubers having 11 mg/100 g DW of carotenoids of which 4.7 mg/100 g DW is represented by β-carotene (Diretto et al., 2007).

Another interesting gene in carotenoid biofortification is the gene encoding 1-deoxyxylulose-5-phosphate synthase (DXS). The DXS enzyme acts in the MEP pathway, upstream of IPP formation, in the plastid isoprenoid pathway (Estevez et al., 2001; Sayre et al., 2011; Ruiz-Sola and Rodríguez-Concepción, 2012), thereby acting also upstream of biosynthesis of a whole range of metabolites depending on this pathway, including tocochromanols (see vitamin E). This approach has been adopted in cassava, tomato and Arabidopsis (Estevez et al., 2001; Enfissi et al., 2005; Sayre et al., 2011). The idea of changing carotenoid content via engineering of a further upstream component proves to be applicable, as shown in tomato, as fruit-specific down-regulation of *DE-ETIOLATED1* (*DET1*) [a light signaling pathway controlling gene (Schafer and Bowler, 2002)], leads to enhancement of both carotenoid and flavonoid levels (Davuluri et al., 2005). Fruit-specific RNAi suppression of an epoxycarotenoid deoxygenase (NCED), a key enzyme in abscisic acid (ABA) biosynthesis, resulted in enhanced lycopene and β-carotene levels (Sun et al., 2012). Strikingly, metabolism of different vitamins could be intertwined, potentially positively influencing their accumulation and stability, as was the case with the combined biofortification of vitamin E and carotenoids in ‘Golden Sorghum’ (Che et al., 2016). This further emphasizes the importance of considering vitamin stability, especially upon long-time storage. In this respect, down-regulation of a lipoxygenase gene (*r9-LOX1*), known to cause carotenoid oxidation (Wu et al., 1999; Blancquaert et al., 2017) in rice endosperm yielded enhanced provitamin A stability in Golden Rice upon storage (Gayen et al., 2015). Suppressing enzymes involved in vitamin breakdown has also been implemented as a metabolic engineering strategy and successfully demonstrated in wheat. Endosperm-specific stimulation of carotenoid biosynthesis by bacterial phytoene synthase was combined with silencing of carotenoid hydroxylase, leading to kernels accumulating up to 500 μg/100 g DW of β-carotene (Zeng et al., 2015).

These strategies are, however, species and likely tissue-specific, as different crops require adjusted engineering approaches. Assessment of their implementation in different agronomically important crops would be a great leap forward (Kang et al., 2017). In this respect, the ability of processing habits to lower vitamin bioavailability should be taken into consideration (Diaz-Gomez et al., 2017a). Interestingly, interventions in provitamin A metabolism resulted in remarkable alterations in crop properties. This has been reported for provitamin A biofortified cassava, achieved by DXS and CTRb (bacterial phytoene synthase) introduction, resulting in prolonged shelf-life upon storage as well as aberrant carbon partitioning causing a significant reduction in dry matter content (Beyene et al., 2018). This further emphasizes the importance of taking all aspects of plant physiology into consideration, not only upon designing but also upon evaluation of biofortified crops.

#### Breeding

Enhancement of provitamin A content in food crops has not been limited to transgenic metabolic engineering approaches, as different breeding projects have also led to successes (Giuliano, 2017; Haskell et al., 2017). Interestingly, studies implementing genome-wide association (GWAS), association analysis and quantitative trait locus (QTL) mapping, pinpoint the factors strongly influencing carotenoid accumulation. Indeed, as maize exhibits a strong natural variation in carotenoid content, germplasm analysis indicated a lycopene cyclase to be the major determinant of the vitamin level (Harjes et al., 2008). QTL analysis of different crops mostly revealed the same genes to be major effectors in carotenoid accumulation, corresponding to those genes also implemented in successful metabolic engineering approaches such as *PSY, LCY*, and *DXS* genes (Giuliano, 2017). Analysis of carotenoid variation could also highlight negative regulators, as was the case for the gene encoding BCH1 (Yan et al., 2010). Molecular techniques have enabled breeding of high vitamin yielding crops. Exemplary cases include biofortified corn (up to 1.5 mg/100 g DW of β-carotene) (Muzhingi et al., 2011; Palmer et al., 2018; Zunjare et al., 2018), cassava (800 μg/100 g DW of β-carotene) (Welsch et al., 2010; Ilona et al., 2017) and sweet potato (400 μg/100 g FW of RAE of provitamin A) (Low et al., 2017). The latter is already reaching almost three million households in Sub-Saharan Africa, thanks to the Sweet Potato for Profit and Health Initiative (SPHI), which aims to provide this orange-fleshed sweet potato (OFSP) to 10 million households (Laurie et al., 2018). Unfortunately, satisfactory variation in rice germplasm to support adequate breeding for enhanced provitamin A content of the endosperm, has not been found (De Moura et al., 2016). A nice overview of achievements in provitamin A biofortified crops is given in a recent review of Giuliano (2017).

### Provitamin A: Major Problems and Future Prospects

The successful creation of provitamin A rich rice, coined Golden Rice, is a good example of a product with great potential, the introduction of which is hampered by regulatory obstructions (Potrykus, 2010, 2017). Indeed, though the potential humanitarian benefit as well as adequate cost-effectiveness of Golden Rice are well known (Stein et al., 2006), current societal perception, strongly following the precautionary principle, has blocked the implementation of Golden Rice for almost two decades. Ingo Potrykus, one of the creators of Golden Rice, has referred to this impediment as ‘a crime against humanity’ (Potrykus, 2010). The rationale behind this, is the calculated amount of Disability-Adjusted Life Years (DALY) (over a million) as well as deaths (over 40 thousand) that could be saved annually by Golden Rice implementation (Potrykus, 2010; De Steur et al., 2017; Wesseler and Zilberman, 2017). The case of Golden Rice holds an important lesson to minimize regulatory obstructions for products of genetic engineering. Satisfactory proof-of-concepts are often difficult to commercialize due to intellectual and tangible property right (Kowalski et al., 2002). When the ultimate goal of a biofortification endeavor goes beyond the academic proof-of-concept, one must thoroughly examine every patent or intellectual property right attached to it. In the case of the Golden Rice project, all licenses -for the technologies involved- have been acquired, enabling free distribution to farmers, provided that the transgenic event is approved (Potrykus, 2017). This was possible, as it is considered a humanitarian project, allowing to be deployed in developing countries by a Humanitarian Use Technology Transfer (HUTT) license. More strikingly, the Golden Rice event GR2-R1 was found to disrupt the native *OsAUX1* (encoding an auxin influx transporter) expression, yielding detrimental consequences for plant growth and development (Bollinedi et al., 2017). This further emphasizes the importance of characterizing the genomic place of insertion and potential influences on growth and development.

Provitamin A is an example of a micronutrient for which major progress has been achieved in biofortification over the last decades (Giuliano, 2017). A substantial part of these accomplishments has been realized via breeding endeavors (Bouis and Saltzman, 2017; Ilona et al., 2017), without the use of genetic engineering and therefore more readily accepted for commercial release (Potrykus, 2017). Focus should now be directed toward proper information of the public on allowing provitamin A rich crops created via GM-technology, so that these can be deployed to decrease VAD in populations which are in need. Moreover, the case of tomato fruit, which naturally contains sufficient lycopene, thus requiring a downstream metabolic engineering intervention to redirect the biosynthetic pathway, is a nice example on how general knowledge of a food crop steers biofortification approaches. Therefore, acquiring a general metabolic engineering strategy is difficult and future research should first be directed to understanding provitamin A biosynthesis within the target crop tissue as well as natural variation in the germplasm thereof. The latter could put breeding strategies forward as a valuable solution to fight VAD. Finally, given the success of breeding strategies in provitamin A biofortification and the natural variation of sexually compatible germplasm they depend on, expanding the available germplasm of a certain crop could have very beneficial impacts.

## Vitamin C – Ascorbate

Ascorbate or L-ascorbic acid (AsA), referred to as vitamin C, is a potent water-soluble antioxidant (Iqbal et al., 2004; Macknight et al., 2017). This molecule is, however, unstable, as it easily deteriorates, being sensitive to heat, alkaline environments and oxygen (Iqbal et al., 2004). Vitamin C *sensu lato* includes all molecules (vitamers) which can be metabolized to form ascorbic acid in human metabolism, including dehydroascorbic acid (Wilson, 2002). Ascorbic acid is a weak sugar acid, related to, and derived from, hexoses (Pohanka et al., 2012).

### Vitamin C Biosynthesis

**FIGURE 3:**
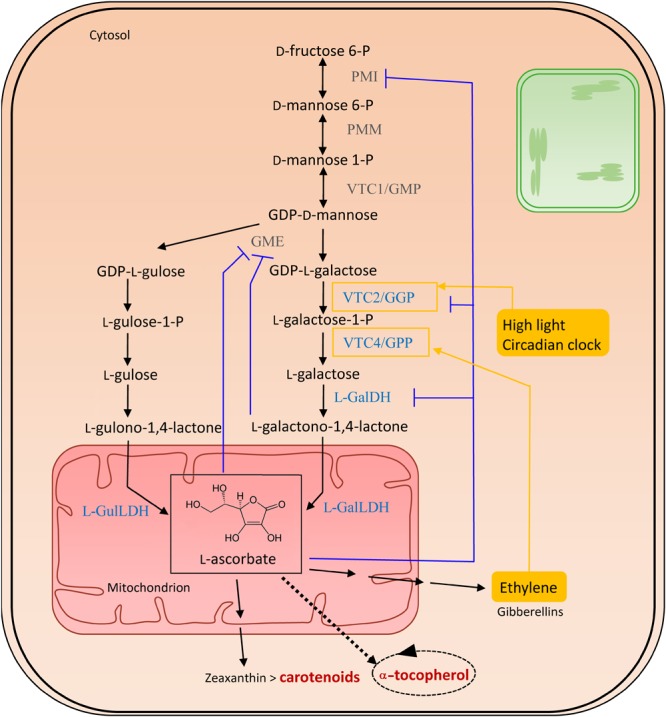
Biosynthesis of vitamin C. The enzymes committed to vitamin C (VTC) biosynthesis are marked in blue. Feedback regulations are illustrated in purple. Filled yellow boxes indicate the external influences on the biosynthesis, regulating enzymes surrounded by a yellow square. Biosynthesis and salvage links to vitamins A and E, in dark red, are indicated with a double and a dashed arrow, respectively. The dashed oval arrow represents the recycling of α-tocopherol, within which ascorbate aids in the detoxification of tocopheroxyl radicals. Abbreviations (in order of appearance in the pathway): PMI, phosphomannose isomerase; PMM, phosphomannose mutase; VTC1/GMP, GDP-D-mannose pyrophosphorylase; GDP-D-mannose, guanosine diphosphate mannose; GME, GDP-mannose-3′,5′-epimerase; VTC2/GPP, GDP-L-galactose-phosphorylase/L-galactose guanylyltransferase; VTC4/GPP, L-galactose 1-phosphate phosphatase; L-GalDH, L-galactose dehydrogenase; L-GalLDH, L-galactono-1,4-lactone dehydrogenase; L-GulLDH, L-gulono-1,4-lactone dehydrogenase.

The sole physiologically significant source of AsA is provided via the Smirnoff-Wheeler pathway, following a route via D-mannose (D-Man) and L-galactose (L-Gal), essentially taking place in the cytosol, with the exception of the final mitochondrial step generating L-AsA (Figure 3) (Ishikawa et al., 2008). Therefore, hexoses need to be directed into D-Man metabolism by phosphomannose isomerase (PMI), followed by the conversion of D-Man-6-P into D-Man-1-P by phosphomannomutase (PMM) (Qian et al., 2007; Maruta et al., 2008). The reversible phosphorylation of D-mannose-1-phosphate (D-Man-1-P) by GDP-D-mannose pyrophosphorylase (GMP/VTC1) results in GDP-D-Man, which is subsequently equilibrated with its epimer, GDP-L-galactose (GDP-L-Gal), through GDP-D-mannose-3,5-epimerase (GME) (Wolucka and Van Montagu, 2007). However, this enzyme can also produce GDP-L-gulose, which occurs in 25% of the epimerization events. This leads to the alternative biosynthesis route, named the L-gulose pathway, which might be species or tissue specific. GDP-Gal is converted to L-galactose-1-Phosphate (L-Gal-1-P) by GDP-L-Gal phosphorylase/L-Gal guanylyltransferase (GGP/VTC2), the first committed and rate-limiting step in the vitamin C biosynthesis pathway (Linster and Clarke, 2008). Both transcription and activity of GGP appear light-regulated, explaining the increase in ascorbate levels in high light conditions. Furthermore, as their diurnal pattern of expression was also observed in constant darkness, GGP is assumed to be under circadian clock control (Dowdle et al., 2007) (Ishikawa et al., 2018). Additionally, *VTC2* is suggested to be controlled by a *cis*-acting upstream open reading frame in high ascorbate conditions (Laing et al., 2015). Several other enzymes are also feedback-inhibited by AsA, including PMI (Maruta et al., 2008), GME (Wolucka and Van Montagu, 2003) and LGalDH (Mieda et al., 2004). In the subsequent step in ascorbate biosynthesis, L-Gal-1-P is hydrolyzed to L-galactose (L-Gal) by L-Gal-phosphate phosphatase (GPP/VTC4) (Conklin et al., 2006), followed by an NAD-dependent oxidation into L-galactono-1,4-lactone (GalL) by L-galactose dehydrogenase (L-GalDH). The last step is yet another oxidation, exerted in mitochondria by the flavin containing L-galactono-1,4-lactone dehydrogenase (L-GalLDH), forming AsA which uses cytochrome c as an electron acceptor (Wheeler et al., 1998; Leferink et al., 2008). This enzyme also shows a diurnal expression pattern (Tamaoki et al., 2003). In the case of the alternative gulose pathway, L-gulono-1,4-lactone is formed, and further converted into AsA by L-GulL dehydrogenase (Wolucka and Van Montagu, 2003). Both GalL and AsA, being low molecular-weight solutes, might cross the outer membrane without the need of a carrier.

### Vitamin C Functions *in planta*

The physiologically active form of vitamin C is its anionic form, ascorbate. The water soluble ascorbate anion (AH-) is a universal player in both enzymatic and non-enzymatic antioxidant defense systems and therefore implicated in a range of processes in plants. Its efficiency as an antioxidant most probably relies on the (relative) stability of its primary oxidation product, the monodehydroascorbate radical (MDA) and moreover, on its capacity to terminate radical chain reactions by spontaneously disproportionating into the non-toxic, non-radical product AsA and dehydroascorbate (DHA) (Noshi et al., 2016).

#### Antioxidant

AsA is of great importance during photosynthesis, firstly because it is capable to donate electrons to PSI and PSII in both normal and stress conditions (Mano et al., 2004; Ivanov, 2014). Moreover it eliminates directly superoxide (O^-^_2_), hydroxyl radicals (^∙^OH) and singlet oxygen (^1^O_2_) coming from photoreduction and photorespiration and aids in the scavenging of hydrogen peroxide being a cofactor of ascorbate peroxidase (APX) in the Asada-Halliwel pathway or Mehler-peroxidase pathway (Foyer and Halliwell, 1976; Shigeoka et al., 2002). The latter pathway, also known as the ascorbate-glutathion cycle (ASC-GSH cycle), involves APX, monodehydroascorbate reductase (MDHAR), dehydroascorbate reductase (DHAR) and glutathione reductase (GR) and is of uttermost importance in the antioxidant defense of plants (Foyer and Halliwell, 1976). Despite the multiple scavenging processes present in plants, lipids still receive the burden of oxidative stress leading to the generation of lipid peroxyl radicals. Clearing thereof is accomplished by α-tocopherols (see vitamin E), which in turn are recycled through the oxidative action of AsA (Davey et al., 2000). In addition, ascorbate, being the cofactor of violaxanthin de-epoxidase (VDE), plays a role in the xanthophyll cycle, as mentioned above in the section of vitamin A, protecting PSII from photoinhibition (Eskling et al., 1997).

#### Development

A wide range of hormone-AsA interactions influence plant physiology. First, AsA is involved as a cofactor of GA3-oxidase and ACC-oxidase in the biosynthesis of gibberellin (GA) and ethylene, respectively (Arrigoni and De Tullio, 2000; Van de Poel and Van Der Straeten, 2014). Second, hormones can also control AsA biosynthesis. In seed tissue, enhanced levels of ABA suppresses activity of NADPH oxidases, the main producers of ROS in seeds (Ishibashi et al., 2017). The resulting decrease of ROS in the aleuron layers inhibits AsA and concomitant GA biosynthesis (Ye et al., 2012). On the other hand, ROS, and more specifically exogenous H_2_O_2_, were shown to enhance expression in imbibed seeds of biosynthesis genes of GA, an essential hormone in seed germination (GA20ox1, GA20ox2, GA20ox3, GA3ox1, and GA3ox2) (Liu et al., 2010; Ye et al., 2012).

Furthermore, AsA was shown to be implicated in sustaining seedling growth. Simultaneous loss of function of two homologs (*vtc2-1 and vtc5-1* or *vtc5*-*2*) encoding the biosynthesis enzyme GGP, results in growth inhibition after cotyledon expansion, followed by bleaching. In later stages of development, ascorbate is required for growth, as the older leaves of the rescued double mutants started to bleach again when transferred back to L-Gal-free medium, the immediate downstream product of these isoforms. Moreover, growth reduction was already observed in the *vtc2* null mutant, in accordance with its low ascorbate level (20%) as compared to wild-type (Dowdle et al., 2007). AsA is also linked with cell expansion and division. Culture experiments showed an increase in ascorbate levels during cell elongation in tobacco, while addition of an ascorbate biosynthesis inhibitor (lycorin) induced cell cycle arrest in G1 in onion root cells (Liso et al., 1984; Kato and Esaka, 1999). This link could partially be attributed to its function as a cofactor of prolyl hydroxylase which converts proline residues in hydroxyproline-rich glycoproteins such as extensins in the cell wall (Fry, 1986; Kerk and Feldman, 1995; De Tullio et al., 1999; Joo et al., 2001; Sanmartin et al., 2007). Moreover, the observation of a depleted level of ascorbate together with an increased activity of ascorbate oxidase (AOX) in the quiescent center (QC) in maize roots are suggestive for a role of ascorbate in the maintenance of QC identity. The concomitant augmented auxin level revealed a regulatory role of the latter on AOX expression (Kerk and Feldman, 1995). Moreover, shoot apical dominance is stimulated by ascorbate (Barth et al., 2006; Kotchoni et al., 2009; Zhang C.J. et al., 2011). Through control of GA and ABA, AsA is also involved in flowering, programmed cell death and senescence (Barth et al., 2006; Kotchoni et al., 2009). AsA and ABA were also shown to influence the expression of senescence associated genes (SAGs) in an antagonistic way (Barth et al., 2006).

Finally, fruit ripening is also related to AsA (Sanmartin et al., 2007). Ascorbate aids in fruit ripening by its counterintuitive site-specific pro-oxidant function. This involves the apoplastic conversion of O_2_ and Cu^2+^ into H_2_O_2_ and Cu^+^, which thereupon combine to generate OH radicals. The presence of the latter results in polysaccharide degradation causing fruit softening (Fry, 1998). In addition, AsA is involved in ethylene biosynthesis (see above), which is essential to induce ripening, and in turn, induces AsA biosynthesis via upregulation of *VTC4* expression (Figure 3) (Ioannidi et al., 2009).

### Vitamin C in Human Health

#### Functions and Pathophysiology of the Deficiency

In human physiology, ascorbate functions as an important scavenger of ROS, such as hydrogen peroxide (Lobo et al., 2010). Importantly, ascorbate is also required as a reducing agent in the conversion of iron from ferric (Fe^3+^) to ferrous (Fe^2+^) oxidation state, thereby aiding in sufficient iron uptake and thus indirectly linked to anemia in case of deficiency (Iqbal et al., 2004; Macknight et al., 2017). Furthermore, vitamin C assists in the metabolism of tryptophan, tyrosine and folate (Iqbal et al., 2004). Moreover, AsA aids in lowering excess cholesterol levels, thereby reducing atherosclerosis (Das et al., 2006; Chambial et al., 2013). This vitamin is also known to function as a cofactor in several reactions such as hydroxylation of muscle carnitine, amidation of several hormones, and the conversion of the neurotransmitter dopamine into norepinephrine (Chambial et al., 2013). Hence, the function of ascorbate is evidently linked to energy metabolism. In collagen biosynthesis, prolyl and lysyl hydroxylases utilize AsA as a enzymatic cofactor (Myllylä et al., 1984; Pimentel, 2003). This explains the pathogenesis of scurvy, a vicious disease, caused by severe vitamin C deficiency, characterized by bleeding gums and eventually leading to edema, jaundice, hemolysis, spontaneous bleeding, neuropathy and death (Leger, 2008). Strikingly, there have been indications that ascorbate supplementation could have a negative impact on tumor development (Cha et al., 2013; Mastrangelo et al., 2018). Moreover, high vitamin C status could prevent or cure several infections (Carr and Maggini, 2017). Evidence indicates that low vitamin C status, though not immediately depicting clinical symptoms, hampers ideal human functioning, as increasing vitamin C uptake is known to be beneficial (Johnston et al., 2006, 2014).

#### Prevalence of Vitamin C Deficiency

Incidence of Vitamin C deficiency is difficult to quantify, as clear deficiency-induced disorders only occur upon very severe ascorbate shortage. Furthermore, there is no consensus on ideal vitamin C intake quantities (Frei et al., 2012; Hickey et al., 2014). Indeed, retrieving an ideal recommended daily intake for vitamin C has been a heavily debated issue, even tackled by Nobel Prize winner Linus Pauling (Pauling, 1974). However, it remains undeniable that increasing the vitamin C status would exhibit positive effects on general human health (Macknight et al., 2017). There is, however, no controversy about the presence of vitamin C deficiency in the general public, despite the infrequency of scurvy. Vitamin C status was reported as being deficient in about 20% of the low-income population of the United Kingdom (Mosdol et al., 2008). Comparable results were obtained by analysis of the north-American population, where smoking and low socio-economic status were identified as risk factors for vitamin C deficiency (Cahill et al., 2009; Schleicher et al., 2009).

#### Vitamin C Sources

Most animals are capable of *de novo* ascorbic acid biosynthesis, given its vital role in their metabolism. However, humans (but also guinea pigs and bats) have lost this privilege due to mutation in the L-gulono-γ-lactone oxidase (*GLO*) gene (cf. L-GulLDH in Figure 3) (Nishikimi et al., 1988; Imai et al., 1998), the evolutionary reason of which has been questioned (De Tullio, 2010). This leaves humans dependent on sufficient dietary ascorbate intake to preserve vital functioning. Fresh (citrus) fruits, tomatoes, broccoli and leafy vegetables are considered excellent sources of vitamin C (Iqbal et al., 2004; Chambial et al., 2013). Unfortunately, ascorbate is prone to deteriorate upon storage or processing, as its content declines upon exposure to heat and oxygen (Lee and Kader, 2000). Vitamin C losses during storage can be decreased via limited exposure to heat and oxygen (Lee and Kader, 2000; Sapei and Hwa, 2014). Though most staples are poor sources of vitamin C, potato and cassava do supply a significant amount of the vitamin to the populations relying on these crop products (Table 1). However, elevating the levels of vitamin C in these crops could deliver additional health advantages.

### Vitamin C Biofortification

#### Metabolic Engineering

Metabolic engineering strategies, aimed at elevating ascorbate levels in a specific crop/tissue, have been deployed by increasing either ascorbate biosynthesis, salvage or altered pathway regulation (Macknight et al., 2017). Interestingly, these approaches possess the ability to increase tolerance to abiotic stresses such as drought, salinity, cold, heat and high light. In ascorbate biosynthesis, the conversion of GDP-L-galactose to L-galactose-1-P, the central step in plant ascorbic acid biosynthesis, carried out by the GDP-L-galactose phosphorylase (GGP, VTC2) enzyme (see Figure 3), is mainly considered as being rate-limiting, thereby a prime target for metabolic engineering approaches (Bulley and Laing, 2016; Macknight et al., 2017). This has been adequately demonstrated in tomato and potato, where introduction of the kiwi and potato *GGP* gene, respectively, yielded an ascorbate increase up to sixfold in tomato fruit and threefold in potato tubers (Bulley et al., 2012). Though other steps in ascorbate biosynthesis have been evaluated in metabolic engineering, GGP remains the most successful (Macknight et al., 2017). Ascorbate salvage on the other hand, the retrieval of ascorbic acid from the oxidized dehydroascorbic acid vitamer, has been tackled using dehydroascorbate reductase (*DHAR*) (Li et al., 2012). Similarly, ascorbate degradation has been engineered via RNAi-mediated downregulation of *AOX* in tomato fruit, resulting in augmented vitamin C levels (Zhang Y.Y. et al., 2011). Furthermore, the Arabidopsis ethylene response factor AtERF98, positively regulating ascorbate biosynthesis, has been implemented in metabolic engineering attempts, as its overexpression in Arabidopsis resulted in enhanced ascorbate levels concomitant with increased salt tolerance (Zhang et al., 2012). This should, however, be approached with caution, as the impact on other aspects of plant metabolism/physiology requires in-depth knowledge of the affected metabolic pathways (Macknight et al., 2017). Moreover, AsA stability should be considered upon evaluation of metabolic engineering strategies. Indeed, after 8 months storage, a drop of vitamin C levels of almost 90% was demonstrated in pasteurized pink guava nectar juice (*Psidium guajava* L.) (Ordonez-Santos and Vazquez-Riascos, 2010). Thus, a metabolic engineering approach combining multiple aspects of ascorbate metabolism including as biosynthesis, recycling, stability and potentially regulation, might prove to yield higher but also stable vitamin C augmentation.

#### Breeding

Given the relatively low increase in ascorbate levels upon metabolic engineering approaches, breeding methods might catch up with these interventions. In pepper (*Capsicum annuum*), which can be considered a rich source of vitamin C, a 2.5-fold variation was observed within the 7 genotypes examined (Geleta and Labuschagne, 2006). The high heritability of this trait indicates a great potential in breeding programs in vitamin C biofortification of pepper. In tomato, transcriptomic analysis of an introgression line exhibiting 4-fold difference in fruit AsA content, pinpointed pectine degradation (particularly pectinesterases) as an important determinant for vitamin C accumulation (Di Matteo et al., 2010; Ruggieri et al., 2015). By QTL mapping of introgression lines, tomato fruit ascorbate levels were also linked to a single nucleotide polymorphism (SNP) near the *MDHAR* genomic region (Sauvage et al., 2014; Bulley and Laing, 2016). Subsequently, analysis of a high ascorbate/carotenoid introgression line enabled identification of an L*-ASCORBATE OXIDASE* allele (*AOX*) as a determinant for AsA levels, the expression of which negatively correlated with vitamin C content (Calafiore et al., 2016). Interestingly, the same study identified an *NCED* allele, to indirectly control AsA accumulation. In apple, a sixfold variation in AsA content found over 28 commercial varieties allowed creation of a mapping population, pinpointing *GGP* alleles as major determinants of fruit vitamin C content (Mellidou et al., 2012). Together, these findings illustrate the vast potential of screening crop germplasms for high vitamin C accumulating varieties, and implement these plants in GWAS and breeding programs.

### Ascorbate: Major Problems and Future Prospects

Given its antioxidant nature and a diversity of potential roles, pathophysiological manifestations are not easily attributable to ascorbate deficiency. This is likely the main cause for the dissent on the ascorbate RDA value, which in turn provokes an underestimation of vitamin C deficiency. Therefore, there is a great need to further underline the tremendous health benefit of improving ascorbate status on a global scale, despite the absence of typical deficiency symptoms. As inherent ascorbate levels in wheat and rice endosperm are negligible (Table 1), metabolic engineering strategies in these tissues might be challenging. However, ascorbate metabolic engineering strategies could be fruitful in helping these crops cope with abiotic stresses. Moreover, metabolic engineering has the potential to convert potato into an ideal medium to deliver sufficient quantities of a potent water-soluble antioxidant, ascorbate, to the population. Future biofortification strategies on the other hand, should, based on the available knowledge on ascorbate function in plant physiology, try to exploit ascorbate accumulation to enable creation of nutritionally enhanced crops with concomitant increased stress tolerance.

## Vitamin E – Tocochromanols

**FIGURE 4:**
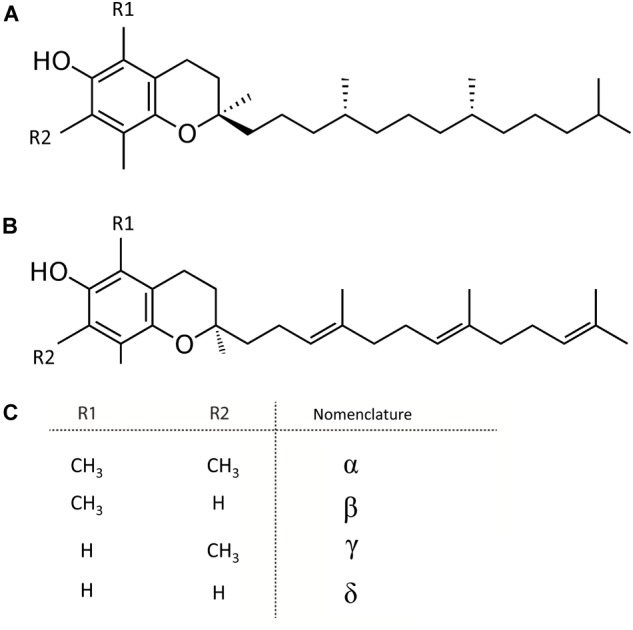
Structure of tocochromanols. **(A)** Tocopherol. **(B)** Tocotrienol. **(C)** Nomenclature of molecules arising from diversity in (R1 and R2 substituents).

Vitamin E or tocochromanols, which includes tocopherols and tocotrienols, are fat-soluble, amphipathic molecules (Colombo, 2010). These molecules consist of a lipophilic isoprenoid chain carrying a polar chromanol ring, providing their amphipathic nature (Figure 4). The molecular structure of these vitamers contains three chiral centers, resulting in 8 stereoisomers of each vitamin E entity (Figure 4). Depending on the substituents on the chromanol ring, both tocochromanols groups exist as α-, β-, γ-, and δ-isomers. Vitamin E molecules are known as potent antioxidants, as they are free radical scavengers, of which α-vitamers are most powerful (Niki and Traber, 2012).

### Vitamin E Biosynthesis

Tocochromanols are synthesized only in the plastids of photosynthetic organisms. While tocopherols are present throughout the plant, tocotrienol is found almost exclusively in seeds and fruits. Both groups and their isoforms occur in different tissues and exert different functions. α-tocopherol resides mainly in the leaves of vascular plants, while γ-tocopherol is the predominant form in seeds (Grusak and DellaPenna, 1999; Abbasi et al., 2007). Indeed, as seen in Arabidopsis, seeds typically exhibit a more pronounced γ-tocopherol contribution to the total tocopherol pool (Gilliland et al., 2006). The precursors of tocochromanols are derived from two different pathways, the shikimate and the MEP pathway, which are also delivering the precursors of the plastidial biosynthesis of folate (B9) and carotenoids (provitamin A), respectively (Mène-Saffrané and Pellaud, 2017).

**FIGURE 5:**
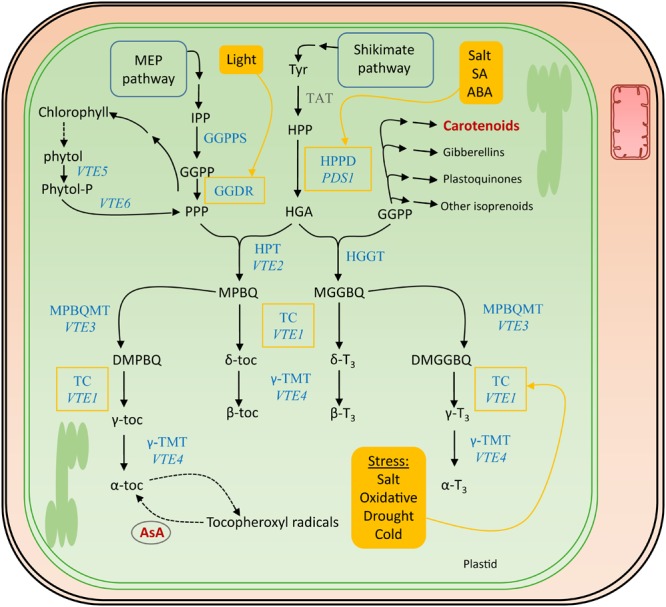
Biosynthesis pathway of vitamin E. The actual enzymes involved in vitamin E biosynthesis, which occurs in the plastids, are marked in blue. The corresponding genes encoding the most important enzymes in *Arabidopsis thaliana* are written in italics. Biosynthesis and salvage links to the other discussed vitamins, in dark red, are indicated with a double and a dashed arrow, respectively. Filled yellow boxes indicate the inducers, regulating enzymes surrounded by a yellow square. Tyr, tyrosine; TAT, tyrosine aminotransferase; HPP, p-hydroxyphenylpyruvic acid; HPPD, HPP dioxygenase; *PDS1, PHYTOENE DESATURATION1;* HGA, homogentisic acid; GGDR, geranylgeranyl diphosphate reductase; HGGT, geranylgeranyl transferase; HPT, homogentisate phytyltransferase; *VTE, VITAMIN E PATHWAY* gene (1–6); GGPP, geranylgeranyl pyrophosphate; PPP, phytyl pyrophosphate; MGGBQ, 6-geranylgeranyl-benzoquinol; MPBQ, 2-methyl-6-phytyl-1,4-benzoquinone; MPBQMT, MPBQ methyltransferase; DMGGBQ, 2,3-dimethyl-6-geranylgeranyl-1,4-benzoquinone; DMPBQ, 2,3-dimethyl-6-phytyl-1,4-benzoquinone; TC, tocopherol cyclase; γ-TMT, γ-tocopherol methyltransferase; α-,β-,γ-,δ-toc, α-,β-,γ-,δ-tocopherol; α-,β-,γ-,δ-T_3_, α-,β-,γ-,δ-tocotrienol; SA, salicylic acid; ABA, abscisic acid.

The polar phenolic p-hydroxyphenylpyruvic acid (HPP), synthesized from tyrosine by tyrosine aminotransferase (TAT) and therefore the shikimate pathway (Figure 5), is used to produce the aromatic ring of the tocochromanols (Figure 4). HPP dioxygenase (HPPD) catalyzes the onset of the actual tocochromanol biosynthesis by converting HPP into homogentisic acid (HGA) after which the pathway bifurcates toward the production of tocopherols and tocotrienols through condensation of two different metabolites bearing the polyprenyl chains (Figure 5).

The MEP pathway delivers the precursors for the biosynthesis of prenyl side chains of tocochromanols, as described for provitamin A biosynthesis (see section “Provitamin A Functions *in planta*”). This branch of tocochromanol biosynthesis utilizes GGPP (geranylgeranyl diphosphate). Interestingly, this product serves as a substrate of multiple enzymes in biosynthesis of different metabolites, including carotenoids, gibberellins, and plastoquinones (Ruiz-Sola et al., 2016). Reduction of GGPP by geranylgeranyldiphosphate reductase (GGDR) yields phytyl diphosphate (PPP) (Gramegna et al., 2018). In the absence of light, PIF3 physically interacts with the promoter of GGDR, down-regulating its expression. Light activation of phytochromes prevents that interaction, leading to transcriptional derepression of the GGDR promotor. The resulting product of GGDR activity, PPP, can be utilized for both tocopherol and chlorophyll biosynthesis (Tanaka et al., 1999). Moreover, PPP is recycled from chlorophyll breakdown, by phytol kinase (VTE5) and phytol-phosphate kinase (VTE6) (Vom Dorp et al., 2015). This was revealed by feeding studies in Arabidopsis which demonstrated the incorporation of labeled phytol in tocopherols in seedlings (Ischebeck et al., 2006). Notably, in ripening fruit tissues, often an important source of tocochromanols, recycling of phytol from chlorophyll breakdown is witnessed to be the predominant PPP source (Gramegna et al., 2018).

Condensation of PPP and HGA by HGA phytyl transferase (HPT/*VTE2*) leads to the formation of 2-methyl-6-phytylbenzoquinol (MPBQ), a step toward creation of tocopherols (Sattler et al., 2004). On the other hand, HGA geranylgeranyl transferase (HGGT) catalyzes the condensation of GGPP with HGA, yielding 6-geranylgeranyl-benzoquinol (MGGBQ), leading toward the formation of tocotrienols (Cahoon et al., 2003; Mène-Saffrané and Pellaud, 2017). These two benzoquinol products, MPBQ and MGGBQ, resulting from HGGT and MGGBQ action, giving rise to tocopherols and tocotrienols, respectively, mark the branch point of tocopherol/tocotrienol biosynthesis. This is illustrated by higher accumulation of tocotrienols in *HGGT*-overexpressing barley (*Hordeum vulgare*) lines, depicting decreased tocopherol levels and therefore relatively unaltered total tocochromanol levels (Chen et al., 2017). Downstream reactions follow a similar pattern for both tocopherols and tocotrienols, as the catalysis is performed by shared enzymes. Cyclization of MPBQ and MGGBQ results in δ-tocochromanols (δ-tocopherol and δ-tocotrienol, respectively), a reaction which is executed by tocopherol cyclase (TC, *VTE1*) (Porfirova et al., 2002; Semchuk et al., 2009). However, MPBQ and MGGBQ can take a different route by methyltransferase reactions (MPBQMT, *VTE3*), resulting in the formation of 2,3-dimethyl-6-phytyl-1,4-benzoquinone (DMPBQ) and 2,3-dimethyl-6-geranylgeranyl-1,4-benzoquinone (DMGGBQ) (Cheng et al., 2003). Cyclization of these products by the aforementioned TC results in the formation of γ-tocochromanols. These γ-tocochromanols and δ-tocochromanols can thereafter be methylated by γ-tocopherol methyltransferase (γ-TMT,*VTE4*) to α-tocochromanols and β-tocochromanols, respectively (Bergmuller et al., 2003).

### Vitamin E Functions *in planta*

#### Scavenger of Lipid Peroxyl Radicals

The most important role of vitamin E *in vivo* is the termination of a chain reaction of polyunsaturated fatty acid (PUFA) free radicals generated by lipid oxidation. Hence, they play a vital role in scavenging lipid peroxyl radicals during germination and early seedling growth. The detrimental decrease in germination potential of TC mutants (*vte1-1*) show they are indispensable to preserve the viability of seeds during seed quiescence, which might explain the elevated level of γ-tocopherol in seeds (Sattler et al., 2004). The upstream biosynthesis mutant *vte2*, which lacks the intermediary DMPBQ, displays difficulties in early seedling development attributable to a decrease in both synthesis and catabolism of lipids as well as an increase in lipid oxidation (Sattler et al., 2004). The few *vte2* plants that survive up to the adult stage display no phenotypical differences from wild type which is explained by a predominant need for tocopherols during early development when essential carbon is recruited from lipid catabolism and gluconeogenesis. At later stages, other antioxidants can mitigate the deficiency of tocopherol-mediated ROS scavenging. Hence, tocopherols and its precursors are important to attenuate lipid peroxidation at specific developmental or stress-related periods (Sattler et al., 2006).

#### Antioxidant, Photoprotectant, and Stress Signaling

The antioxidant function of tocopherols is supported by the ascorbate-glutathione cycle which recycles tocopheroxyl radicals produced during the reaction of tocopherols with lipid peroxyl radicals. Moreover, tocochromanols are, albeit with a lower rate constant than carotenoids, quenchers of singlet oxygen (^1^O_2_) (Kaiser et al., 1990). Up to 120 molecules of ^1^O_2_ can be neutralized by one molecule of α-tocopherol through resonance energy transfer (Fahrenholtz et al., 1974). Related to their scavenging capability, tocochromanols have strong photoprotective properties. When exposing the alga *Chlamydomonas* to high light, the inhibition of HPP-dioxygenase led to decreased levels of α-tocopherol and concomitantly, to the inactivation of PSII (Trebst et al., 2002). Addition of synthetic, cell-wall permeable, short-chain tocopherol derivatives could partly restore photosynthesis, hence tocopherols are implicated in the maintenance of PSII function, supplemental to the photoprotective function of NPQ (Trebst et al., 2002; Havaux et al., 2005; Kruk et al., 2005). Thus, tocochromanols together with carotenoids and zeaxanthin are the major protectors of PSII against photoinhibition, as they control D1 protein degradation by scavenging singlet oxygen molecules in PSII, and they also protect the whole thylakoid membrane against photooxidative stress, by controlling lipid peroxidation (Trebst et al., 2004). In young leaves of a carotenoid mutant devoid of zeaxanthin, high light stress induced accumulation of tocopherols, conferring tolerance to the mutant, suggesting overlapping functions for these antioxidants (Havaux et al., 2000; Golan et al., 2006). Recently, it was found that an oxidation product, tocopherol quinone, can function as an indicator of oxidative stress, transforming into a signal for programmed cell death upon severe stress. Herewith, the plant protects itself from propagation of stress from the infection point (Li Y. et al., 2008). Moreover, defense-related genes were expressed at higher levels in *vte2* plants in response to an increase in peroxidized lipids, suggesting that tocopherol plays a role in gene regulation and modulation of defense responses (Sattler et al., 2006). In this respect, α-tocopherol was found to be important in the mitigation of salt and heavy metal stresses (Jin and Daniell, 2014). In rice, expression of the *VTE1* gene was induced by high salt, H_2_O_2_, drought and cold, while overexpression led to increased tolerance to salt stress (Ouyang et al., 2011). Conversely, tocopherol deficient Arabidopsis mutants displayed similar phenotypes as wild types under most stress conditions (high light, salinity and drought) applied (Maeda et al., 2006). Hence, in case of tocopherol shortage, other antioxidants can take over its role in stress, yet, vitamin E is an additive value in harsh conditions.

#### Membrane Fluidity and Phloem Transport

Besides their role as lipophilic antioxidant, tocochromanols also act as important structure-stabilizing agents of membranes (Wang and Quinn, 1999). Their concentration in the chloroplast is most probably tightly regulated as a low concentration of α-tocopherol, comparable with the physiological plastidial concentration, seemed to have an important effect on membrane stability during freezing (Hincha, 2008). On that account tocopherols help, together with other components, to maintain the fluidity and thus the function of photosynthetic membranes.

Furthermore, tocopherols have been suggested to play a role in the regulation of photoassimilate export and thus be involved in carbohydrate metabolism, source-sink relationships and growth (Sattler et al., 2003; Hofius et al., 2004). In that respect, a tocopherol cyclase mutant of maize *sucrose export defective1 (sxd1)* suggested the link between the tocopherol pathway and carbohydrate metabolism as it accumulated carbohydrates in leaves (Russin et al., 1996). The same was observed in *StSXD1* RNA interference knockdown lines in potato, but surprisingly not in the *vte1* mutant in Arabidopsis, suggesting species-specific differences to tocopherol reduction or a possible additional role of tocopherol in signal transduction (Sattler et al., 2003; Hofius et al., 2004; Li Y. et al., 2008). The biosynthesis mutants *vte2* and, to a lesser extent, *vte1* revealed inhibition of photoassimilated carbon transport at low temperatures and thus indicated a crucial role of tocopherol in low-temperature adaptation. Cold, non-freezing conditions resulted in a dramatic growth reduction and seed production in these mutants due to structural changes in the phloem parenchyma transfer cells induced by callose deposition and thus leading to reduced photoassimilate export. Lipid peroxidation and photoinhibition were not intensified in *vte2*, leading to the conclusion that vitamin E function in phloem transport might be more important than its photoprotective role. Apparently the intermediate redox-active DMPBQ can compensate for the absence of tocopherols as the phenotype of *vte1* is not as pronounced as of *vte2* (Maeda et al., 2006).

### Vitamin E in Human Health

#### Function and Onset of Deficiency

As antioxidants, the different E-vitamers play an important role in neutralizing ROS and inhibiting membrane peroxidation, very much like they do in plants. Due to their amphipathic character, they reside in the membranes, where they perform their peroxyl scavenging function (Brigelius-Flohe, 2009). The main role of these vitamers is to maintain the integrity of long-chain polyunsaturated fatty acids, thereby ensuring their bioactivity (Traber and Atkinson, 2007). Vitamin E deficiency can induce changes in phospholipid composition of membranes, possibly leading to reduced fertility (Infante, 1999). Indeed, vitamin E, together with the micronutrient selenium, has been suggested to serve as a supplement to treat male infertility (Keskes-Ammar et al., 2003). Though tocopherols, predominantly α-tocopherols, are present at higher levels in the human body, significance of tocotrienols should not be neglected (Sen et al., 2006; Colombo, 2010). Indeed, tocotrienols have shown to be effective in inhibiting proliferation of cancers (Aggarwal et al., 2010; Kannappan et al., 2012), albeit that the ability to impede tumorigenesis also has been documented for tocopherols (Li et al., 2011). Vitamin E is also known to have a positive effect on human health by negatively influencing the occurrence of atherosclerosis and cardiovascular diseases (Mathur et al., 2015). Furthermore, vitamin E, α-tocopherol in this case, was shown to delay the development of Alzheimer’s disease in patients (Dysken et al., 2014; La Fata et al., 2014). Indeed, vitamin E deficiency aggravates or even induces neurodegenerative disorders (Berman and Brodaty, 2004; Wysota et al., 2017). Hence, vitamin E has been proposed as a therapeutic agent for Alzheimer’s disease (Ibrahim et al., 2017). Vitamin E deficiency can impair cognitive functioning, particularly in elderly people (Ortega et al., 2002), which could be explained by aberrant brain energy metabolism, also known to be associated with thiamin deficiency (Sang et al., 2018; Strobbe and Van Der Straeten, 2018) and phospholipid composition (McDougall et al., 2017).

#### Global Vitamin E Status

Vitamin E deficiency, though not often identified as the causative agent of pathophysiological disorders, is known to be highly prevalent in different populations. Strikingly, a vast majority of the US population is characterized by insufficient intake of dietary α-tocopherol (Maras et al., 2004), the predominant dietary source of vitamin E (Chun et al., 2006). Assessment of vitamin E intake in the French and Italian population, indicated a significant prevalence of suboptimal vitamin E levels (Polito et al., 2005). Interestingly, vitamin E status of the Italian population appeared superior compared to the French, which could be attributed to the typical dietary habits in the Italian culture (see below). More recently, approximately one-fourth of the Korean population (in the Seoul metropolitan area) was found to be vitamin E deficient, based on plasma α-tocopherol levels (Kim and Cho, 2015). Furthermore, analysis of blood α-tocopherol levels, confirmed the presence of vitamin E deficiency in many developing countries (Dror and Allen, 2011).

#### Sources of Vitamin E

Good plant-based sources of dietary (bioactive) vitamin E, in some cases interpreted as supply of α-tocopherol, are fat and oily products such as dried nuts, seeds and almonds (Maras et al., 2004). Tomatoes, avocadoes, spinach, and olives deliver a significant portion of vitamin E (Chun et al., 2006). Though vegetables are generally not a good source of vitamin E (α-tocopherol), soybean and dark leafy greens do exhibit relatively high tocochromanol content. This could explain the rather high vitamin E status of the Italian population (Polito et al., 2005), given the consumption of vitamin E-rich vegetable oil in this region (Huang and Sumpio, 2008). Indeed, the traditional Mediterranean diet has been associated with health benefits, similar to vitamin E, such as reduced incidence of cardiovascular diseases and decreased lipid oxidation (Fito et al., 2007). Starchy, energy-rich staples on the other hand, can be considered rather poor contributors to dietary the vitamin E supply (Table 1).

### Vitamin E Biofortification

#### Metabolic Engineering

Biofortification to enhance vitamin E content in different crops has been successfully deployed over the last decades (Mène-Saffrané and Pellaud, 2017). To understand the rationale behind these strategies, one must first consider the different biological activities of the E-vitamers. As mentioned above, in many cases, α-tocopherol is considered the most potent, bioactive E-vitamer, as confirmed in a rat fetal resorption assay (Bunyan et al., 1961; Mène-Saffrané and Pellaud, 2017). Interestingly, important vitamin E sources such as vegetable oils (soybean, corn, canola and palm) contain a high ratio (up to 10:1) of γ-tocopherol over α-tocopherol (Eitenmiller, 1997). As α-tocopherol was determined to be ten times more bioactive as compared to γ-tocopherol, the idea arose to design metabolic engineering approaches shifting this ratio toward an enhanced relative α-tocopherol content (Shintani and DellaPenna, 1998). However, this objective needs to be justified by assessing the bioavailability as well as storage stability of these vitamers. Indeed, no compelling differences in bioavailability of these E-vitamers were found (Reboul et al., 2008; Reboul, 2017). Unfortunately, α-tocopherol appears less stable in storage, as it reacts faster with peroxy radicals, confirmed by the higher instability of α-tocopherol compared to γ-tocopherol in storage of camelina (*Camelina sativa*) oil (Abramovic et al., 2007). Although this issue should not be neglected, the higher bioactivity of the α-tocopherol vitamer could outweigh this disadvantage. Introduction of a γ-tocopherol methyltransferase (γ-TMT) (Figure 5) (Tewari et al., 2017), catalyzing the addition of the required methyl group to form α-tocopherol from γ-tocopherol (Figures 4, 5), was therefore conducted. This strategy was proven successful in Arabidopsis, where the α/γ-tocopherol ratio was completely reversed in favor of α-tocopherol accumulation in seeds overexpressing the γ*-TMT* gene (Shintani and DellaPenna, 1998; Mène-Saffrané and Pellaud, 2017). This strategy has been implemented in several crops, including corn (Zhang L. et al., 2013), soybean (*Glycine max*) (Arun et al., 2014) and lettuce (*Lactuca sativa* L.) (Cho et al., 2005). Theoretically, the biological activity of the crop vitamin E pool can be increased up to 10-fold by this strategy (Mène-Saffrané and Pellaud, 2017). In rice endosperm, ectopic γ*-TMT* expression yielded no significant change in α-tocopherol content, explained by low γ-tocopherol levels, yet significantly altered tocotrienol levels, in favor of α-tocotrienol (Zhang G.Y. et al., 2013). Interestingly, implementation of this metabolic engineering approach, yielding higher α-tocopherol content in alfalfa leaves (*Medicago sativa*), coincided with a delayed leaf senescence phenotype as well as enhanced tolerance to osmotic stress (Jiang et al., 2016). As this strategy does not greatly influence accumulation of the absolute tocochromanol content, applicability is confined to crops accumulating higher levels of E-vitamers with lowered bioactivity, such as γ-tocopherols and δ-tocopherols. Furthermore, generalization of E-vitamers into absolute values of ‘bioactivity’ could prove to be difficult. Indeed, different vitamers could exhibit different potencies in a whole range of biological functions, but without a single vitamer being omnipotent. This is indicated by the observed higher ability of γ-tocopherol to reduce 8-isoprostane [oxidative stress marker (Elfsmark et al., 2018)] (Jiang et al., 2002; Jiang and Ames, 2003). Assigning a universal (vitamin E) bioactivity to a specific vitamer could miss identifying its full biological potential. Moreover, the typical accumulation of γ-tocopherols witnessed in seeds (Sattler et al., 2004; Gilliland et al., 2006) (and therefore contributing to the vitamin E content of oils), might hint at its physiological importance *in planta*. Fortunately, no aberrant growth and fertility have been reported in the γ*-TMT*-engineered biofortified crops, indicating that the altered tocopherol ratio has marginal effects on plant growth and development (Mène-Saffrané and Pellaud, 2017).

Besides redirection of tocopherol homeostasis toward a more satisfactory vitamer composition, increase of (absolute) vitamer content has been tackled in metabolic engineering approaches (Cahoon et al., 2003). Engineering the *HGGT* gene, catalyzing the committed step in tocotrienol biosynthesis (Figure 5), resulted in an increase in total tocochromanol content of maize kernels and up to 18-fold enhancement in tocotrienol accumulation (Dolde and Wang, 2011). Furthermore, engineering HPPD, a key enzyme in the biosynthesis of the tocochromanol precursor HGA (Figure 5), generated a massive accumulation of tocotrienols, provided that prephenate dehydrogenase (shikimate pathway) was also engineered to ensure sufficient flux toward tyrosine (Rippert et al., 2004). Building further on this approach, high tocochromanol accumulating soybean was created via additional introduction of *HPT* and *GGDR* (see Figure 5) (Karunanandaa et al., 2005). However, biofortification approaches should not neglect tocochromanol stability, as vitamin E levels were shown to halve in freeze-dried fortified apple upon 6 months storage (Cortes et al., 2009). Further details on the different strategies employed in biofortification of crops toward higher vitamin E content have been elaborated by Mène-Saffrané and Pellaud (2017).

#### Breeding

From the perspective of plant breeders, an interesting amount of variation in vitamin E content has been observed in different agronomical important crops (Mène-Saffrané and Pellaud, 2017). In rice, total kernel vitamin E content was found to vary up to threefold in different Malaysia-grown varieties (Shammugasamy et al., 2015). Similarly, a study in canola, which is important for oil production and therefore tocochromanol delivery, identified *VTE3* and *PDS* as important determinants of tocopherol content, based on screening of 229 accessions (Fritsche et al., 2012). Moreover, a measured variation of almost sixfold in maize kernel α-tocopherol content enabled conducting a GWAS wherein a *HGGT* gene, a prephenate dehydratase paralog [participating in tyrosine biosynthesis (El-Azaz et al., 2016)] and a tocopherol cyclase were recognized to contribute to tocotrienol content (Lipka et al., 2013). The same study further confirmed the link between *γ-TMT* alleles and α-tocopherol content. Interestingly, more recent GWAS in maize revealed many significant QTL loci, attributed to genes harboring novel activities as well as participating outside the tocopherol pathway (Diepenbrock et al., 2017; Wang et al., 2018). In conclusion, this is a nice example of GWAS and assignment of candidate genes to the identified QTLs to pinpoint potential factors for novel metabolic engineering approaches.

### Tocochromanols: Major Problems and Future Perspectives

The case of tocochromanols, comprising tocopherols and tocotrienols, is a good example on how simplifying these distinct groups of molecules to their collective term ‘vitamin E’ can be misleading. As previously mentioned, the bioactivity of E-vitamers is diverse. However, bioactivity alters depending on which tocochromanol-related process is utilized to assess it. Moreover, there is no one-to-one relationship between a certain vitamer and a given function. One could therefore argue that grouping tocochromanols into one group of ‘vitamin E’ is incorrect. This notion becomes more important given the existence of different metabolic engineering approaches aimed at altering E-vitamer ratios (e.g., increasing α-tocopherol/γ-tocopherol ratio) while keeping total tocochromanol levels intact (γ-TMT-engineering). Similarly, bioavailability as well as (storage) stability should not be neglected. Moreover, whether engineering approaches are based on altering tocochromanol ratio (e.g., via γ-TMT-engineering) or enhancing total tocochromanol content (e.g., *HGGT*-engineering), the impact on plant growth and development should be closely monitored. Finally, seeds, being an important target for metabolic engineering approaches, often depict a typical tocochromanol signature (Sattler et al., 2004), related to their function therein, which could be disrupted upon engineering approaches. Future research should therefore further unravel the *in planta* role of the different vitamin E entities. Similarly, the pathophysiological significance of the different vitamers in humans should be thoroughly examined.

## Intertwining of Vitamin Metabolism and Its Significance in Multi-Biofortification

A simultaneous increase of several micronutrients in a particular crop/tissue, referred to as multi-biofortification, is a powerful means to tackle MNM. This strategy aims at obtaining adequate levels of multiple micronutrients in a single staple crop, which is massively consumed by the local population in need. Such endeavor might encounter synergistic but also potentially detrimental effects, due to micronutrient interactions. Taking the example of the antioxidant ascorbate, protection of components sensitive to oxidative damage (e.g., carotenoids) is expected, thereby contributing to their accumulation as well as stability upon storage, an advantage which could also be expected from the combination with vitamin E. Furthermore, the ascorbate-glutathione pathway is needed in the ‘detoxification’ of tocopheroxyl radicals in vitamin E salvage (Szarka et al., 2012). In addition, ascorbate is known to ameliorate iron uptake in humans (Iqbal et al., 2004). Consequently, ascorbate biofortified crops could also aid in combatting iron deficiency indirectly. Similarly, provitamin A and vitamin E biofortification have shown to be positively affect one another (Che et al., 2016; Muzhingi et al., 2017). In the example of biofortified sorghum, the raised level of vitamin E, obtained by genetic engineering, enhanced provitamin A stability (Che et al., 2016). Interestingly, a synergistic interrelationship between ascorbate and vitamin B9 (folates) has been proposed, justified by their coextensive increase during germination (Liu et al., 2017). This study also proposes that folates (vitamin B9) biosynthesis counteracts vitamin E biosynthesis by its competition for the precursor GTP. Competition for precursors could prove to have a substantial influence on vitamin metabolism, considering the fact that vitamin E biosynthesis requires precursors from shikimate and MEP pathways, which are also required in the folate and provitamin A pathways, respectively. Conversely, folates are proposed to aid in maintaining high ascorbate content, as they contribute in supplying NADPH to the cell, which could support adequate ascorbate salvage (Gorelova et al., 2017; Liu et al., 2017). Moreover, DXS activity, which has been enhanced in different metabolic engineering approaches aimed at augmenting plant provitamin A content, requires active B1 vitamer cofactor (thiamin pyrophosphate) for its functioning (White et al., 2016) and is also required in the biosynthesis of tocochromanols. This nicely illustrates how different vitamins are part of a potentially strong network of interactions in plant as well as in human metabolism. This aspect certainly deserves proper consideration upon evaluation of novel biofortification strategies (Strobbe and Van Der Straeten, 2018). Furthermore, certain environmental influences could alter the accumulation of multiple vitamins, illustrated by the light-dependent accumulation of both provitamin A and tocochromanols (Cruz et al., 2018; Gramegna et al., 2018). This aspect can therefore be considered upon setting light conditions in vertical farming projects (Bantis et al., 2018).

Last but not least, biofortification could have the beneficial ‘side-effect’ of enhancing tolerance to abiotic stresses, as reported in metabolic engineering approaches enhancing plant ascorbate content (Macknight et al., 2017). This is particularly important given the increased exposure to abiotic stresses, but also to biotic stresses crops will have to face as a result of climate change (Cheeseman, 2016).

## Conclusion

Vitamin biofortification of food crops holds the potential to alleviate the global burden of vitamin deficiencies (Blancquaert et al., 2017; Garcia-Casal et al., 2017; Jiang L. et al., 2017; Martin and Li, 2017; Van Der Straeten et al., 2017; Garg et al., 2018). In doing so, staple crops will play a predominant role, as they hold the impressive capability to deliver cheap calories to populations in need and have the potential to be nutritionally enhanced via metabolic engineering or breeding approaches. Both conventional breeding and metabolic engineering should coexist in the battle against vitamin deficiencies, thereby reciprocally strengthening their potential. Molecular breeding techniques such as GWAS promise to facilitate enhancement of crop vitamin content whilst uncovering potential new determinants in vitamin accumulation in the particular crop/tissue, subsequently applicable in new engineering approaches. In some cases, downregulation of genes impeding vitamin accumulation is advised (see provitamin A biofortification). Here, metabolic engineering strategies utilizing genome-editing techniques such as the CRISPR/Cas system are promising, especially considering they might suffer less from regulatory issues blocking their commercialization (Potrykus, 2017), in cases where no transgenes are introduced. However, this technology still faces crop-specific limitations toward the maximal vitamin enhancement possible. Therefore, a combination with metabolic engineering strategies employing transgenes, is advisable, in which CRISPR/Cas technology could still be utilized to allow specific T-DNA insertion the genome position of interest.

When using a biofortification approach, several aspects should be considered, including bioavailability, bioactivity, stability and impact on crop yield and/or physiology. Bioavailability, bioactivity and stability can be addressed by examination of these properties on the specific biofortified crop, and targeted by specific strategies to confer these properties to the crop product [e.g., engineering toward more stable folates in rice (Blancquaert et al., 2015), or engineering toward more potent α-tocopherol (Shintani and DellaPenna, 1998)]. Assessment of biofortification interventions influencing plant physiology (and thereby yield) requires in-depth analysis and knowledge of micronutrient metabolism as well as *post hoc* examination of plant physiology in field conditions.

Given their potential to provide sufficient micronutrients, (multi-)biofortified crops are a crucial piece of the puzzle in eradicating micronutrient deficiencies on a global scale. Moreover, biofortified crops are already contributing to sustainable food security in a time of increasing global demographic pressure and climate change. Last but not least, they hold great potential to contribute even more to maintaining a healthy world population into the future, provided that novel approaches to biofortification are embraced.

## Author Contributions

All authors listed have made a substantial, direct and intellectual contribution to the work, and approved it for publication.

## Conflict of Interest Statement

The authors declare that the research was conducted in the absence of any commercial or financial relationships that could be construed as a potential conflict of interest.
